# Levels, sources, and risk assessment of PAHs residues in soil and plants in urban parks of Northwest China

**DOI:** 10.1038/s41598-022-25879-8

**Published:** 2022-12-12

**Authors:** Nuerla Ailijiang, Naifu Zhong, Xiaolong Zhou, Anwar Mamat, Jiali Chang, Shuangyu Cao, Zhenyu Hua, Nanxin Li

**Affiliations:** 1 Key Laboratory of Oasis Ecology of Education MinistryCollege of Ecology and Environment, Xinjiang University, Urumqi, 830017 People’s Republic of China; 2 Xinjiang Jinghe Observation and Research Station of Temperate Desert Ecosystem, Ministry of Education, Urumqi, 830017 People’s Republic of China; 3 Institute of Quality Standards and Testing Technology for Agro-Products, Xinjiang Academy of Agricultural ScienceLaboratory of Quality and Safety Risk Assessment for Agro-Products (Urumqi), Ministry of AgricultureKey Laboratory of Agroproducts Quality and Safety of Xinjiang, Urumqi, 830091 People’s Republic of China; 4 School of Chemical Engineering and Technology, Xinjiang University, Urumqi, 830017 People’s Republic of China; 5 Division of Environmental Engineering, School of Chemistry, Resources and Environment, Leshan Normal University, Sichuan, 614000 People’s Republic of China

**Keywords:** Environmental monitoring, Environmental impact

## Abstract

Polycyclic aromatic hydrocarbons (PAHs) will be ingested by people through different ways to threaten their health during play, so the environmental quality of the park directly affects the health of tourists and residents. Using eight typical parks in Urumqi in Northwest China as the study area, we used GC–MS to detect the PAHs content in the park surface soil and 10 common plants in the park in different seasons. The results showed that the content of PAHs in park soil in the summer was 5–6 times that in the winter, and the monomer PAHs in some park soil sampling points were higher than the soil pollution risk screening value. And the contamination level at these sampling sites was also higher compared to other sampling sites. In summer, the plants with high PAHs content in leaves are short herbs, while in winter, they are tall arbors. The PAHs of the park soil are mainly composed of high-cyclic aromatic hydrocarbons, and are mainly of traffic origin. The proportion of low-ring aromatic hydrocarbons in the winter was significantly higher than that in the summer. The source of PAHs in plants in summer is similar to that in soil, but the source of PAHs in plants in winter is more complex. The toxicity equivalent concentration method values of soil PAHs in South Park, Zhiwu Park, Shihua Park and Toutunhe Park were higher than that in other parks. The lifetime carcinogenic risk (ILCRs) values of some sampling points in these four parks in the summer were relatively high. The average ILCRs of adults and children in all parks reached a low-risk level in summer. The carcinogenic risk in children is much higher than that of adults.

## Introduction

Polycyclic aromatic hydrocarbons (PAHs) refer to a type of persistent organic pollutants composed of two or more benzene rings. They have attracted much attention in the field of environmental protection owing to their "three causes" effects^1^. Among them, 16 types of PAHs have been included in the priority list of pollutants by the U.S. Environmental Protection Agency (EPA)^2^. All human production activities such as fossil fuel combustion, motor vehicle exhaust emissions, coke and asphalt production, generate PAHs^3^. PAHs can enter plants through plant leaves or settle into the soil from the atmosphere, and then migrate, metabolize, and accumulate in plants through plant roots, thereby threatening human health through the food chain^4^. In addition, soil, as an important environmental medium, is a storage and transfer station for PAHs in the natural environment, which bears more than 90% of the environmental load of PAHs. The amount of PAHs entering the human body from soil is higher than that from other environmental media, such as air and water^5^.

In recent years, research on PAHs has mostly focused on the analysis and determination of PAH content in crops, farmland soils, and soils around cities, or on the risk assessment of PAHs in the urban atmosphere in different seasons to humans^6–8^. However, there are few studies on PAHs in urban park soils. In addition, studies have reported that plants can promote the degradation and transformation of PAHs in the soil through root exudates, and the leaves of plants can effectively accumulate PAHs in the atmosphere through the effects of gas diffusion and dry–wet sedimentation^9,10^. The study of PAHs in common plants in parks can provide a more detailed understanding of the source and spatial distribution of PAHs in parks.

Urumqi is an important node city of the "the Belt and Road" strategy, an important industrial, cultural, political, economic, scientific, technological and transportation center in Xinjiang, and an important economic center in Western China, with a population of 4.054 million and the green area of the park is 3.06 × 10^3^ hm^2^, the per capita park green area is only 16.14 m^2^, which is far lower than the world average. In the process of playing, PAHs are ingested by human through hand and mouth, inhalation, and skin contact, which threaten their health^11^. The environmental quality of a park directly affects the health of tourists and residents. Therefore, it is necessary to conduct research on the pollution of PAHs in soil and plants in Urumqi Park and investigate the law of seasonal changes.

In this study, eight typical parks in Urumqi City were used as the study area. Samples were collected in the summer and winter to determine PAH residues in the surface soil of the park. Ten common plants in the park were selected to determine the PAHs in the leaves and roots of the plants. Combined with the results of the determination of the concentration of PAHs in the soil, we studied in detail the residual characteristics, sources, and seasonal changes of PAHs in the area. Then, according to the soil health risk assessment method, the health risks and ecological risks of PAHs residues in the soil of the park were identified. As such, we provide the scientific basis for decision-makers to formulate relevant management measures to ensure human health and ecological environment safety.

## Results and discussion

### PAHs content level

As shown in Table 1, according to the screening value of soil pollution risk in the standard for construction land (GB36600-2018), the value of BaP at sampling point 3 of Zhiwu Park in the soil of the park in summer is 0.944 mg/kg, which is greater than the screening value of 0.55 mg/kg. The values of DahN at sampling point 3 of Zhiwu Park and sampling point 3 of Toutunhe Park are 1.469 mg/kg and 0.615 mg/kg, respectively, which are greater than the screening value of 0.55 mg/kg, whereas BAP and DahN mainly originate from vehicle exhaust emissions, such as gasoline and diesel combustion^12^. This shows that the traffic congestion near Zhiwu Park and Toutunhe Park in summer needs to be reasonably controlled. The concentrations of PAHs in other soil sampling points in the summer and winter were lower than the screening value for soil pollution risk.

**Table 1:** The content of PAHs in soil and plants in Urumqi city parks.

PAHs	Ring number	Concentration (mg/kg)												
		Summer						Winter						GB36600-2018
		Soil		Plant (leaf)		Plant (root)		Soil		Plant (leaf)		Plant (root)		
		Range value	Average value	Range value	Average value	Range value	Average value	Range value	Average value	Range value	Average value	Range value	Average value	
Nap	2	0.005–0.012	0.007	0.008–0.016	0.011	0.006–0.012	0.008	0.003–0.044	0.010	0.0028–0.132	0.047	0.006–0.008	0.007	25
Ace	3	0.0002–0.009	0.002	0.0005–0.002	0.001	0.0006–0.001	0.001	0.0004–0.028	0.004	0.007–0.033	0.012	0.0006–0.002	0.001	–
Acy	3	0.0002–0.021	0.006	0.0002–0.002	0.001	0.0005–0.013	0.003	0.0003–0.014	0.003	0.003–0.016	0.007	0.0006–0.015	0.004	–
Flu	3	0.0008–0.035	0.010	0.003–0.013	0.007	0.003–0.005	0.004	0.002–0.047	0.009	0.020–0.104	0.040	0.004–0.006	0.005	–
Phe	3	0.009–0.384	0.168	0.016–0.093	0.052	0.012–0.032	0.022	0.009–0.462	0.087	0.060–0.194	0.149	0.017–0.038	0.023	–
Ant	3	0.0008–0.237	0.035	0.001–0.105	0.021	0.001–0.028	0.007	0.0009–0.083	0.015	0.003–0.013	0.006	0.001–0.004	0.002	–
Fla	4	0.012–0.671	0.232	0.010–0.051	0.022	0.003–0.014	0.009	0.004–0.586	0.099	0.017–0.051	0.032	0.004–0.016	0.009	–
Pyr	4	0.004–0.035	0.197	0.005–0.047	0.020	0.004–0.013	0.008	0.003–0.471	0.075	0.012–0.028	0.022	0.004–0.015	0.008	–
BaA	4	0.012–0.671	0.199	0.007–0.034	0.017	0.003–0.017	0.011	0.0009–0.316	0.044	0.003–0.006	0.004	0.004–0.020	0.013	5.5
Chy	4	0.006–0.516	0.216	0.010–0.048	0.020	0.007–0.016	0.011	0.003–0.382	0.065	0.009–0.025	0.016	0.006–0.021	0.011	490
BbF	5	0.013–1.254	0.548	0.018–0.179	0.054	0.014–0.109	0.063	0.001–0.445	0.068	0.003–0.011	0.006	0.015–0.134	0.052	5.5
BkF	5	0.008–0.641	0.316	0.004–0.091	0.020	0.004–0.041	0.020	0.001–0.241	0.036	0.002–0.005	0.003	0.002–0.048	0.019	55
BaP	5	0.008–0.944	0.260	0.004–0.040	0.011	0.006–0.023	0.014	0.0008–0.290	0.040	0.001–0.003	0.002	0.004–0.027	0.012	0.55
InP	5	0.011–2.458	0.490	0.011–0.106	0.025	0.009–0.066	0.035	0.0008–0.287	0.040	0.001–0.004	0.003	0.005–0.057	0.022	5.5
DahN	6	0.008–1.469	0.312	0.005–0.069	0.014	0.005–0.038	0.020	0.001–0.238	0.036	0.001–0.004	0.003	0.003–0.041	0.016	0.55
BghiP	6	0.002–3.233	0.306	0.004–0.025	0.014	0.002–0.016	0.008	0.0001–0.086	0.011	0.001–0.008	0.001	0.003–0.013	0.008	–
Σ_7_PAH	–	0.093–2.171	2.341	0.089–0.562	0.161	0.052–0.297	0.175	0.009–2.171	0.328	0.024–0.058	0.037	0.050–0.344	0.145	–
Σ_16_PAH	–	0.135–12.427	3.304	0.153–0.905	0.311	0.115–0.377	0.245	0.033–3.941	0.644	0.182–0.625	0.356	0.094–0.446	0.212	–

Σ_7_PAHs: 7 kinds of carcinogenic PAHs (BaA, Chr, BbF, BkF, BaP, InP, DahN); Σ_16_PAHs: 16 kinds of priority control PAHs.

**Figure 1:**
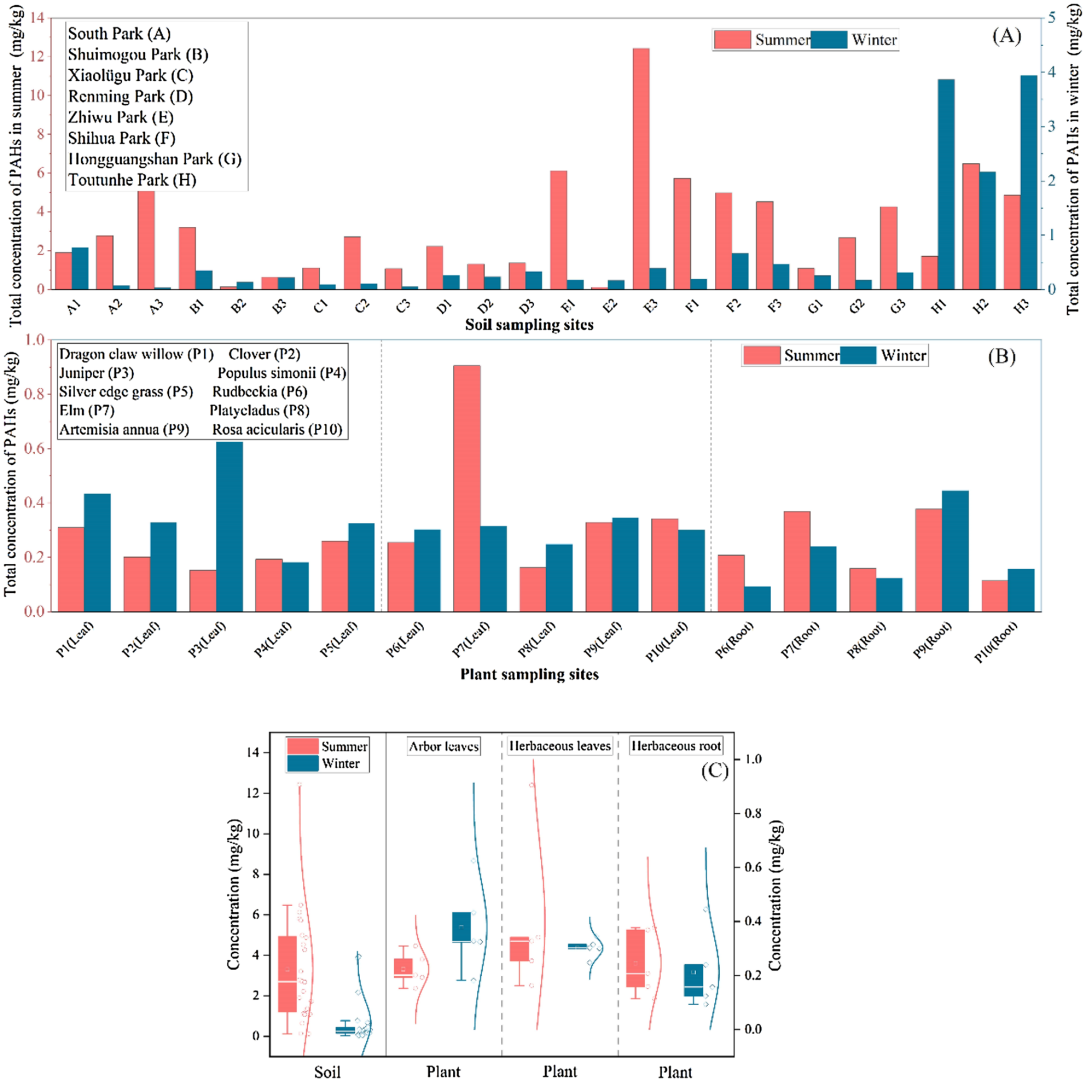
Total concentration distribution of PAHs in park soil and plants.

PAH-contaminated soil can be divided into four levels: no pollution (< 0.200 mg/kg), slight pollution (0.200–0.600 mg/kg), medium pollution (0.600–1.000 mg/kg) and serious pollution (> 1.000 mg/kg)^13^. As we can see from Fig. 1A, in the summer, the sampling point No. 1 in Shuimogou Park and the sampling point No. 2 in Zhiwu Park are pollution-free, the sampling point No. 3 in Shuimogou Park is moderately polluted, and the total concentr Shuimogou sampling ation of PAHs in other park soil Shuimogou sampling points is greater than 1.000 mg/kg, reaching the degree of serious pollution. The highest pollution point was the sampling point No. 3 in Zhiwu Park. The large difference in concentration between different the sampling points in the Zhiwu Park and Shuimogou Park may be caused by factors such as the distance between the sampling points and the road and the dumping of garbage^14^. Among the soil samples in the summer, 91.6% were more than moderately polluted, and only 8.4% were pollution-free. This is because the park has a large passenger flow, especially in the summer, and it is located adjacent to the main traffic road. Traffic volume is large, and traffic emissions are one of the main sources of urban PAHs^15^. In the soil of the winter park, the sampling point No. 1 of South Park and sampling point No. 2 of Shihua Park were moderately polluted. The total concentration of PAHs in the three sampling points of Toutun Park was greater than 1.000 mg/kg, reaching the level of serious pollution. The most polluted point was the sampling point No. 3. Some other sampling points were slightly polluted, and some were pollution-free. Toutun Park is located near the Diwopu Airport. Due to the re-planning and reconstruction of the park in recent years, the exhaust emissions of large trucks and the wear of tires during the reconstruction process have greatly increased the accumulation of PAHs in the park^16^. Shihua Park is located near Midong District. There are numerous chemical plants of petrochemical companies, plastic factories, which generate a large number of PAHs during the production and processing of products. The South Park is located in a downtown area with heavy traffic and many nearby residential areas. Compared with other parks, there are more man-made emission sources near these three parks. In winter, 58.3% of soil samples were polluted, of which 20.8% were moderately polluted or above, and 41.7% were pollution-free. Compared with the summer, the proportion of contaminated samples decreased.

As shown in Table 2, the average order of the soil PAHs in each functional area in summer is traffic area > industrial area > park area > rural area > commercial area, which is consistent with the research by Min et al.^17^. The park is located adjacent to the main traffic road. Compared with the rural and commercial areas, the park area has a larger passenger and vehicular traffic flow. Compared with the traffic area, the traffic volume in the park area is small, and industrial activities such as coke oven gas, organic chemical industry, petroleum industry, steelmaking, and ironmaking in the industrial area generate a large amount of PAH emissions^17^. Conversely, plant leaves in the park absorb PAHs from atmospheric deposition and accumulate PAHs, such that the PAHs entering the soil of the park through atmospheric deposition is greatly reduced. Compared with other regions, the content of soil PAHs in the Urumqi park area in the winter is similar to that in Beijing and Xi'an park area, which is slightly higher than that in the Yinchuan and Fuzhou park areas. This is because Urumqi, Beijing and Xi'an have a large population, and heating in the winter generates more PAHs. The average value of PAHs in the summer park area was 5–6 times that in winter. This is consistent with the results of Fangfang et al.^18^, who determined that the PAH content in individual sampling sites in the park area in winter is higher than that in summer. This may be owing to climatic conditions and location of the sampling site^18^. In addition, at the end of 2019, a new type of coronavirus (COVID-19) was discovered in China, and there were outbreaks all parts of the world. To curb the COVID-19 epidemic, outdoor activities (transportation, industry, entertainment, etc.) in many countries were restricted^19^. This also includes the city of Urumqi. Outdoor activities were not resumed until September 2020, when the outbreak was controlled in Urumqi. And the restriction of outdoor activities potentially reduces the accumulation of PAHs in the soil in winter 2020. Overall, the PAH pollution of soil in the park area of Urumqi City is relatively serious and should be considered.

**Table 2:** The content of PAHs in soil and plants in different regions.

Area	Soil (mg/kg)		References	Area	Plant (mg/kg)		References
	Range value	Average value			Range value	Average value	
Urumqi city transportation area (summer)	2.149–15.709	8.171	^17^	Nanjing campus	0.224–1.098	0.660	^45^
Urumqi City Commercial Area (Summer)	0.669–2.338	1.228	^17^				
Urumqi industrial zone area (summer)	0.741–15.347	6.435	^17^	Guangzhou scenic area	0.460–1.304	0.773	^46^
Rural district of Urumqi city (summer)	0.331–2.752	1.742	^17^				
Yinchuan city park area	0.122–0.373	0.231	^47^	Changsha traffic area	3.66–11.13	6.676	^26^
Fuzhou PARK AREA	0.054–0.385	0.222	^49^				
Xi'an park area	0.362–1.336	0.591	^50^	Qingdao agricultural district	0.078–0.421	0.223	^20^
Beijing park area	0.066–6.867	0.460	^48^				
Urumqi city park area (summer)	0.135–12.427	3.304	This research	Urumqi city park area (summer)	0.033–3.941	0.644	This research
Urumqi city park area (winter)	0.033–3.941	0.644	This research	Urumqi city park area (winter)	0.094–0.625	0.308	This research

The PAH content of plants in the South Park of Urumqi is shown in Table 1. Comparing plant leaves and roots, we can see that whether it is summer or winter, the proportion of Σ_7_PAHs in Σ_16_PAHs is always higher than that of leaves. This is because Σ_7_PAHs are mainly composed of 4–6 ring aromatic hydrocarbons, and the absorption of roots from soil is the main absorption pathway of high-ring PAHs. Low-ring PAHs are the result of the combined effect of leaf absorption from the atmosphere and root absorption from the soil^20^. In addition, the proportion of Σ_7_PAHs in plant leaves in the winter to the concentration of Σ_16_PAHs is considerably different from that in plant leaves and roots in summer and roots in winter. This is because plant leaves mainly absorb PAHs in the atmosphere^21^, and PAHs in the atmosphere are more susceptible to the impact of surrounding emission source than soil. Due to the control of the epidemic, vehicle transportation restrictions led to a reduction in the proportion of high-ring PAHs in the winter.

As shown in Fig. 1B, the highest total concentration of PAHs in the leaves of park plants in summer was Elm leaves, followed by Rosa acicularis leaves and Artemisia annua leaves. These herbaceous plants are relatively short and grow close to the ground, as a result the content of PAHs in their leaves is greatly affected by ground dust^22^. The highest total concentration of PAHs in plant leaves in winter parks is in Juniper leaves, followed by Platycladus leaves and Dragon claw willow leaves. Because these plants belong to arbor plants, the plants are relatively high and the leaves are located far away from the ground, the PAHs content in the leaves is easily affected by PAHs in the atmosphere. In winter, due to heating, the combustion of coal and biomass mainly produces tricyclic PAHs, which are volatile and easily transferred to the atmosphere^23^. In addition, Juniper and Platycladus are evergreen plants in all seasons, and their leaves do not easily fall off. Compared with other plants, they are more likely to accumulate PAHs. Pine needles have a large specific surface area, high surface wax content, wide distribution, and easy collection. They have been used by many foreign environmental scientists to monitor and evaluate organic pollutants in the environment^24^. We can also observe from Fig. 1C that the total concentration of PAHs in 70% of the plant leaf samples was greater in the winter than in the summer. This is owing to volatilization through stomata caused by the rising temperature in the summer. PAHs mainly migrate from leaves to the atmosphere^25^. The content of PAHs in the roots of Clover and Silver edge grass was higher in different seasons. It is speculated that this may be related to the structure of the plant. These two herbaceous plants may be used to repair soil pollution caused by PAHs in the soil^26^. In the study by Yuanyuan et al.^10^, it was found that legume alfalfa and gramineous perennial ryegrass exhibited a higher removal rate of soil PAHs, which was mainly because plants promoted the joint degradation of PAHs in soil by indigenous microorganisms^10^.

Table 2 shows a comparison of the PAH content of plants in this study and other regions. The range of total PAHs in summer plants in Urumqi parks is 0.033–3.941 mg/kg, which is higher than the change range of PAHs in plants considered by Dong Ruibin and others in the range of 0.020–1.000 mg/kg^26^. The concentration value is close to the Nanjing campus area and Guangzhou scenic area, but much lower than the Changsha traffic area. Studies have shown that when a car is driving at a non-uniform speed, the exhaust contains more PAH pollutants, and the PAH content in the exhaust particulate matter also increase relatively^27^. In addition, our results were slightly higher than that in Qingdao agricultural area. This is because the research conducted by Wei et al.^27^ is farmland soil, which is located at the edge of the city and is far away from the city center. Although there are pollution sources such as industry and traffic around some sampling points, these are relatively limited, such that the content of PAHs in plants is close to other regions with relatively low urbanization and industrialization. The average total PAH content of plants in the park area in the summer was twice that in the winter, which is consistent with the PAH content in the soil. This may be correlated with climatic conditions and control of the epidemic.

### PAHs monomer component characteristics

According to the number of rings in PAHs, the 16 types of PAHs are divided into 2–3 low-ring aromatic hydrocarbons (LMW) and 4–6 high-cyclic aromatic hydrocarbons (HMW)^12^. Among them, the 2-ring PAHs are mainly derived from the leakage of source oil, while the 3-ring and 4-ring PAHs are mainly derived from the combustion of coal and biomass, the 5-ring PAHs are mainly derived from the combustion of gasoline, and the 6-ring PAHs are mainly derived from the combustion of diesel.

**Figure 2:**
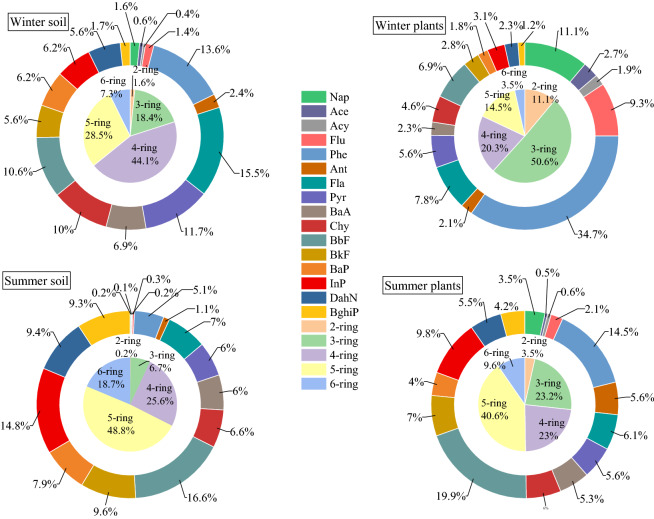
Single component of PAHs in soil and plants and the proportion of different ring numbers.

As shown in Fig. 2, BbF, BkF, InP, and DahN accounted for a higher proportion in the summer soil. BbF mainly originates from the combustion of gasoline, BkF mainly originates from industrial coal, and InP and DahN mainly originate from automobile exhaust^12,28^. The order of the proportion of the number of different PAHs was 5-ring PAHs > 4-ring PAHs > 6-ring PAHs > 3-ring PAHs > 2-ring PAHs. It is speculated that the main source of PAHs in the summer park soil may have originated from gasoline combustion and traffic emissions^16^.

Fla, Phe, Pyr, and BbF accounted for a higher proportion of the soil PAHs in the winter. Fla mainly originates from the combustion of coal and petroleum and can also originate from garbage incineration. Phe mainly originates from the process of coking, which is the process of decomposing and distilling coal to produce coke, and Pyr mainly originates from industrial coal^29^. The order of the proportions of the number of different PAHs was 4-ring PAHs > 5-ring PAHs > 3-ring PAHs > 6-ring PAHs > 2-ring PAHs. Compared with the summer, the proportion of the 3rd and 4th rings in the winter has increased significantly, and the 3rd and 4th rings of PAHs are mainly derived from the combustion of coal and biomass, which is caused by heating in the winter. In addition, it can be found that the contribution of HMW is higher in both summer and winter, which may be owing to the volatility of LMW^23^.

We can observe from Fig. 2 that the higher proportions of summer plants were BbF, Phe, InP, and BkF. Barring Phe, the main proportion of PAHs was similar to the proportion of PAHs in summer park soil, indicating that the proportion of different PAHs in plants is greatly affected by soil. Phe accounts for a relatively small proportion in the summer soil, but a larger proportion in plants. This is because, due to its small molecular weight, Phe is easily volatilized from the soil when the temperature is high in the summer and is thereby absorbed by plant leaves^30^. The order of the proportion of the number of different PAHs was 5-ring PAHs > 3-ring PAHs > 4-ring PAHs > 6-ring PAHs > 2-ring PAHs. Similar to summer soils, high-ring PAHs accounted for a higher proportion, but compared with summer soils, the proportion of LMW increased. This is because low-molecular-weight PAHs have 2–3 rings, which are mainly gaseous and are easily absorbed by plants.

Phe, NaP, Flu, and Fla accounted for a higher proportion of plants in the winter. Among them, Flu mainly originates from coking, and NaP mainly originates from coke oven products and oil spills^30^. The order of the proportion of the number of different PAHs was 3 ring PAHs > 4 ring PAHs > 5 ring PAHs > 2 ring PAHs > 6 ring PAHs. Compared with the summer, the proportion of low-ring PAHs in winter plants and soil has increased significantly, which may be caused by the burning of large amounts of coal and organisms for heating in winter in the north. In winter, the proportion of HMW in plant roots and leaves is different, and the proportion of HMW in root is higher than that in leaves. This is because the roots are underground, and it is easier to absorb high-ring PAHs from the soil than leaves. In addition, the proportion of LMW in plants in both winter and summer was higher than that in the soil. This is because foliar absorption is the main mechanism by which plants accumulate PAHs^31^.

### PAHs source analysis

The feature ratio method is often used to identify the source of PAHs in the environment^32,33^. LMW/HMW > 1 represents the crude oil source, LMW/HMW < 1 represents high-temperature pyrolysis; Ant/(Ant + Phe) < 0.1 represents the crude oil source, Ant/(Ant + Phe) > 0.1 represents the combustion source; BaP/BghiP ratio > 0.6 means traffic emission source, less than 0.6 means non-traffic emission; BaA/(BaA + Chy) < 0.2 characterizes crude oil source; 0.2 < (BaA/BaA + Chy) < 0.35 is used to characterize the combustion of coal, vegetation, etc.; finally (BaA/BaA + Chy) > 0.35 is usually used to characterize the combustion of petroleum and fossil fuels^32,34^.

**Figure 3:**
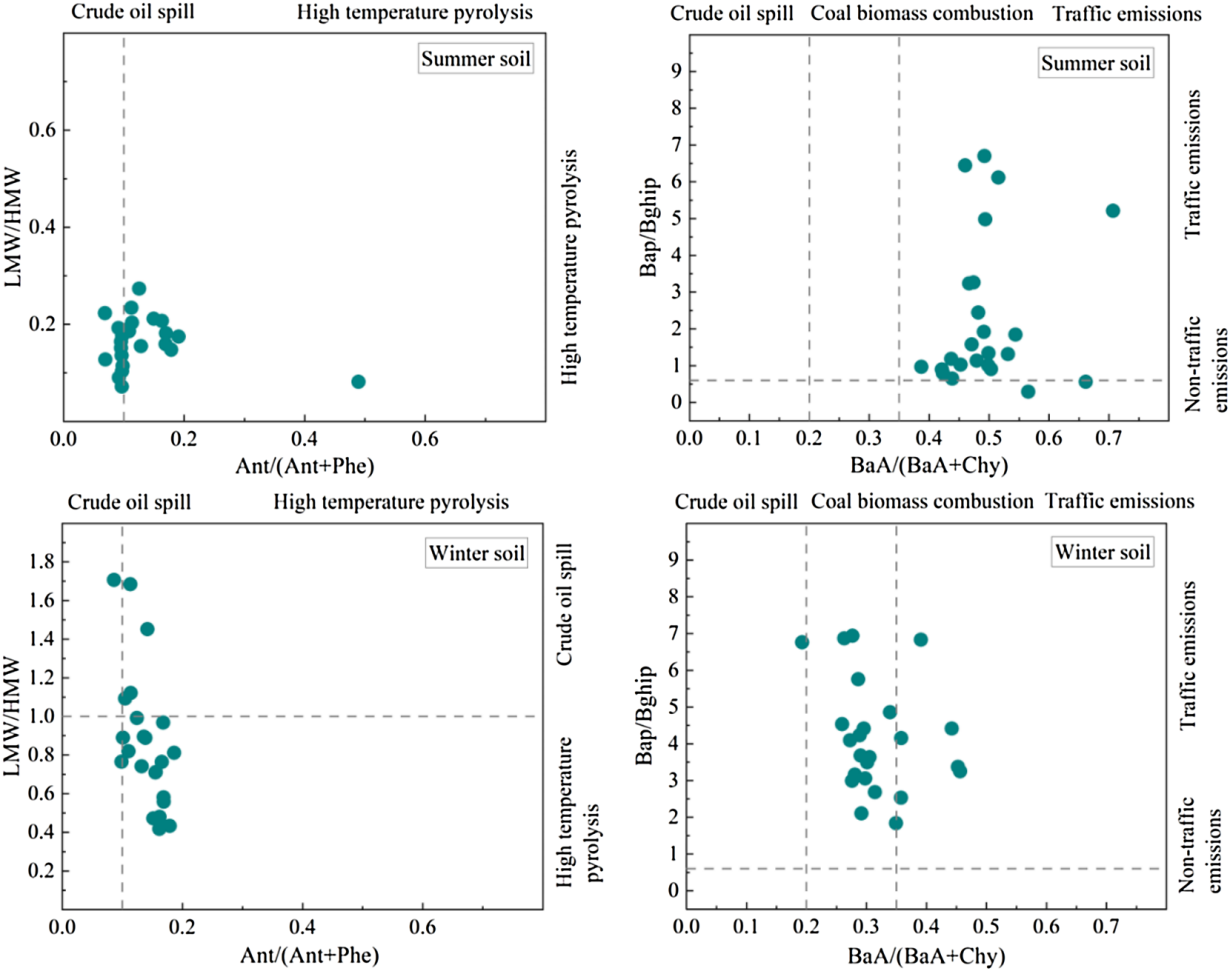
Source analysis of soil PAHs.

As shown in Fig. 3, the ratio of LMW/HMW in summer soil was less than 1, 54.2% of the Ant/(Ant + Phe) ratio was greater than 0.1, and 91.7% of the BaP/BghiP ratio was greater than 0.6. All BaA/(BaA + Chy) ratios were greater than 0.35. We can infer from this that the PAHs in the soil of Urumqi City Park in the summer mainly originate from traffic emissions, that is, the combustion of crude oil fuels such as gasoline and diesel.

In winter, 79.2% of the LMW/HMW ratio in the soil was less than 1, 95.8% of the Ant/(Ant + Phe) ratio was greater than 0.1, the BaP/BghiP ratio was greater than 0.6, and 75.0% of the BaA/(BaA + Chy) ratio was between 0.2 and 0.35. We can infer from this that the PAHs in the soil of Urumqi city parks in winter mainly originate from high-temperature sources, including incomplete combustion of coal, grass, and other biomass and fossil fuels.

**Figure 4:**
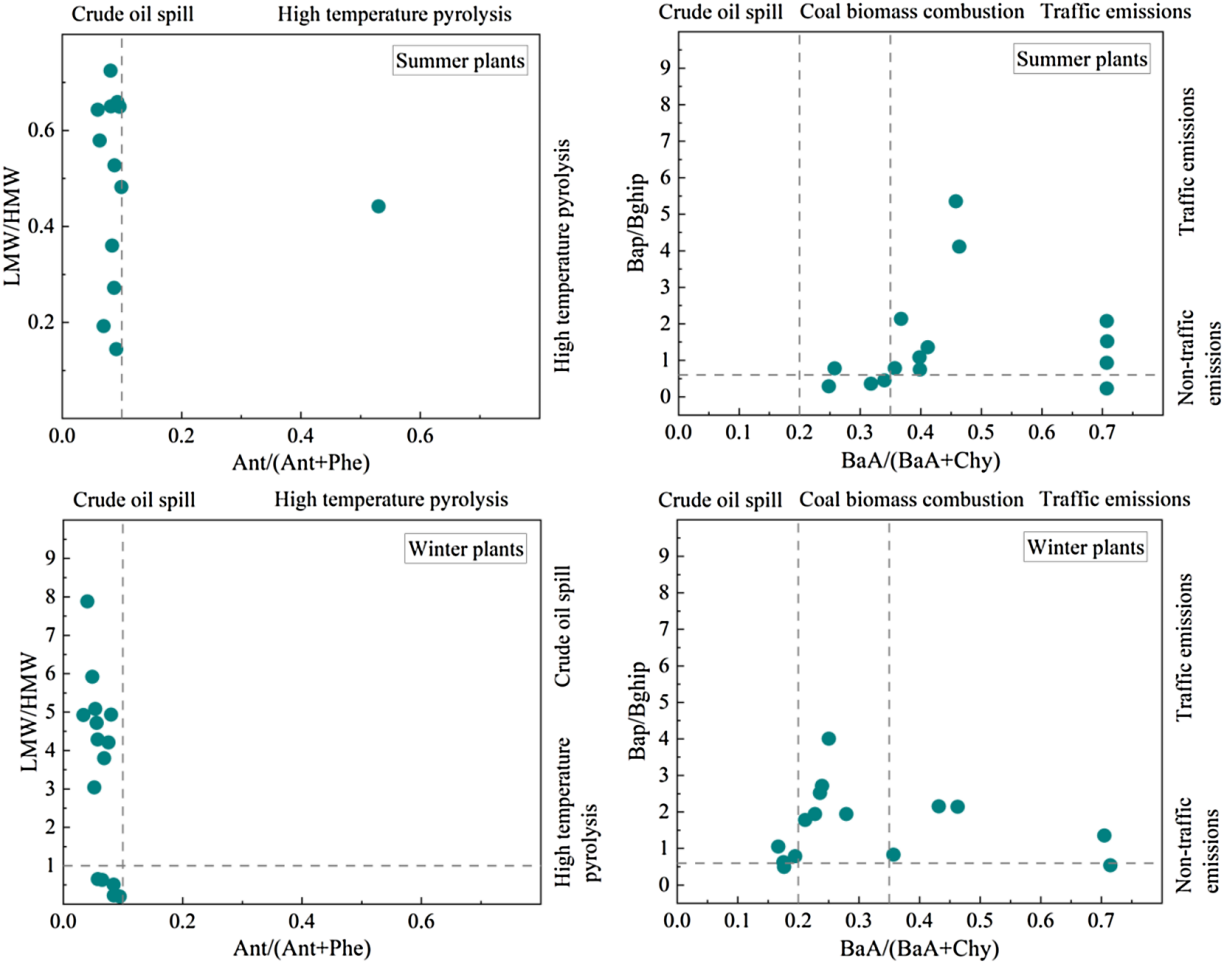
Source analysis of plant PAHs.

As shown in Fig. 4. The LMW/HMW ratios of summer plants were all less than 1, 93.3% of the Ant/(Ant + Phe) ratios were less than 0.1, 73.3% of the BaP/BghiP ratios were greater than 0.6, and 73.3% of the BaA/(BaA + Chy) ratios were greater than 0.35. From this, we can infer that the PAHs in plants of Urumqi park in the summer are similar to those in the soil, and they mainly originate from traffic emissions.

In winter plants, 66.6% of the LMW/HMW ratios were greater than 1, all the ratios of Ant/(Ant + Phe) were less than 0.1, 86.6% of the BaP/BghiP ratios were greater than 0.6, and 33.3% of the BaA/(BaA + Chy) rations were less than 0.2, 33.3% were between 0.2 and 0.35, 33.3% were greater than 0.35. We can infer from this that the source of PAHs in park plants in Urumqi in winter is more complicated, and the leakage of crude oil, burning of coal biomass, and traffic emissions all contribute to a certain extent.

### PAHs risk assessment

Table 3 displays the toxic equivalent concentrations of PAHs in the soil of parks in Urumqi. The TEQ range of PAHs in the summer soil of the park was 0.024–2.937 mg/kg, with an average of 0.733 mg/kg, while the average TEQ of Σ_7_PAHs was 0.729 mg/kg, accounting for 99.5% of the Σ_16_PAHs, and the TEQ of the remaining PAHs only accounts for 0.5%, which shows that Σ_7_PAHs are the primary contributing factors to the toxic effect of the PAHs in the summer soil of the park. The contributions of different monomer PAHs to the total TEQ were as follows: DahN (42.8%) > BaP (35.6%) > BbF (7.5%) > InP (6.7%) > BkF (4.3%) > BaA (2.8%) > Chy (0.3%). Further, 25% of the total TEQ value of PAHs in the soil in Urumqi Summer Park is higher than the standard value of 1 mg/kg given by the World Health Organization (WHO)^35^. These sampling point mainly originate from South Park, Zhiwu Park, Shihua Park and Toutunhe Park. Therefore, direct or indirect contact with the soil of these four parks in the summer cause a certain risk of toxic effects to humans.

**Table 3:** Toxicity equivalent concentration of PAHs in the soil of parks in Urumqi.

PAHs	TEF_Bap_	TEQ (mg/kg)			
		Summer soil (mg/kg)		Winter soil (mg/kg)	
		Range value	Average value	Range value	Average value
Nap	0.001	4.6 × 10^–6^ to 1.2 × 10^–5^	6.9 × 10^–6^	3.0 × 10^–6^ to 4.4 × 10^–5^	1.0 × 10^–5^
Ace	0.001	2.4 × 10_–7_ to 9.7 × 10^–6^	2.0 × 10^–6^	4.4 × 10^–7^ to 2.8 × 10^–5^	4.1 × 10^–6^
Acy	0.001	2.3 × 10^–7^ to 1.7 × 10^–5^	5.7 × 10^–6^	3.2 × 10^–7^ to 1.4 × 10^–5^	2.6 × 10^–6^
Flu	0.001	8.5 × 10^–7^ to 3.5 × 10^–5^	1.0 × 10^–5^	1.2 × 10^–5^ to 4.7 × 10^–5^	9.3 × 10^–6^
Phe	0.001	8.9 × 10^–6^ to 4.4 × 10^–4^	1.7 × 10^–4^	9.5 × 10^–6^ to 4.6 × 10^–4^	8.7 × 10^–5^
Ant	0.01	8.4 × 10^–6^ to 2.3 × 10^–3^	3.5 × 10^–4^	8.9 × 10^–6^ to 8.2 × 10^–4^	1.5 × 10^–4^
Fla	0.001	4.4 × 10^–6^ to 5.0 × 10^–4^	2.3 × 10^–4^	3.7 × 10^–6^ to 5.9 × 10^–4^	9.9 × 10^–5^
Pyr	0.001	3.9 × 10^–6^ to 4.9 × 10^–4^	1.9 × 10^–4^	2.5 × 10^–6^ to 4.7 × 10^–4^	7.5 × 10^–5^
BaA	0.1	1.2 × 10^–3^ to 6.7 × 10^–2^	1.9 × 10^–2^	9.4 × 10^–5^ to 3.2 × 10^–2^	4.4 × 10^–4^
Chy	0.01	6.3 × 10^–5^ to 5.1 × 10^–3^	2.2 × 10^–3^	2.6 × 10^–5^ to 3.8 × 10^–3^	6.4 × 10^–4^
BbF	0.1	1.3 × 10^–3^ to 0.125	5.4 × 10^–2^	1.4 × 10^–4^ to 4.4 × 10^–2^	6.8 × 10^–3^
BkF	0.1	7.8 × 10^–4^ to 6.4 × 10^–2^	3.1 × 10^–2^	1.0 × 10^–4^ to 2.4 × 10^–2^	3.5 × 10^–3^
BaP	1	7.9 × 10^–3^ to 0.943	0.259	7.6 × 10^–4^ to 0.289	3.9 × 10^–2^
InP	0.1	2.3 × 10^–2^ to 7.6 × 10^–2^	4.9 × 10^–2^	7.9 × 10^–5^ to 2.9 × 10^–2^	3.9 × 10^–3^
DahN	1	0.186–0.458	0.312	1.2 × 10^–3^ to 2.6 × 10^–2^	3.6 × 10^–2^
BghiP	0.1	9.4 × 10^–4^ to 2.5 × 10^–3^	3.0 × 10^–3^	1.1 × 10^–6^ to 8.6 × 10^–4^	1.1 × 10^–4^
Σ_7_PAH	–	0.024–2.9	0.729	0.002–0.654	0.095
Σ_16_PAH	–	0.024–2.937	0.733	0.003–0.656	0.096

Σ_7_PAHs: 7 kinds of carcinogenic PAHs (BaA, Chr, BbF, BkF, BaP, InP, DahN); Σ_16_PAHs: 16 kinds of priority control PAHs.

The TEQ range of PAHs in the winter soil was 0.003–0.656 mg/kg, with an average value of 0.096 mg/kg; the average TEQ of Σ_7_PAHs was 0.095 mg/kg, accounting for 99.0% of the Σ_16_PAHs, and the TEQ of other PAHs only accounts for 1.0%, and similar to summer soil, Σ_7_PAHs are almost all contributing factors to the toxic effect of PAHs in the winter soil. The contribution of different monomer PAHs to the total TEQ was in the following order: BaP (41.8%) > DahN (37.8%) > BbF (7.2%) > BaA (4.6%) > InP (4.2%) > BkF (2.7%) > Chy (0.7%). The total TEQ value of PAHs in the soil in Urumqi winter is lower than the standard value of 1 mg/kg given by the WHO. Therefore, direct or indirect contact with Urumqi parks soil in winter will not cause toxic effects to humans.

The lifetime carcinogenic risk (ILCRs) less than or equal to 10^–6^ are considered to be basically negligible lifetime cancer risk; ILCRs between 10^–6^ and 10^–4^ indicate low risk; ILCRs greater than 10^–4^ indicate high potential health risks^36^.

**Figure 5:**
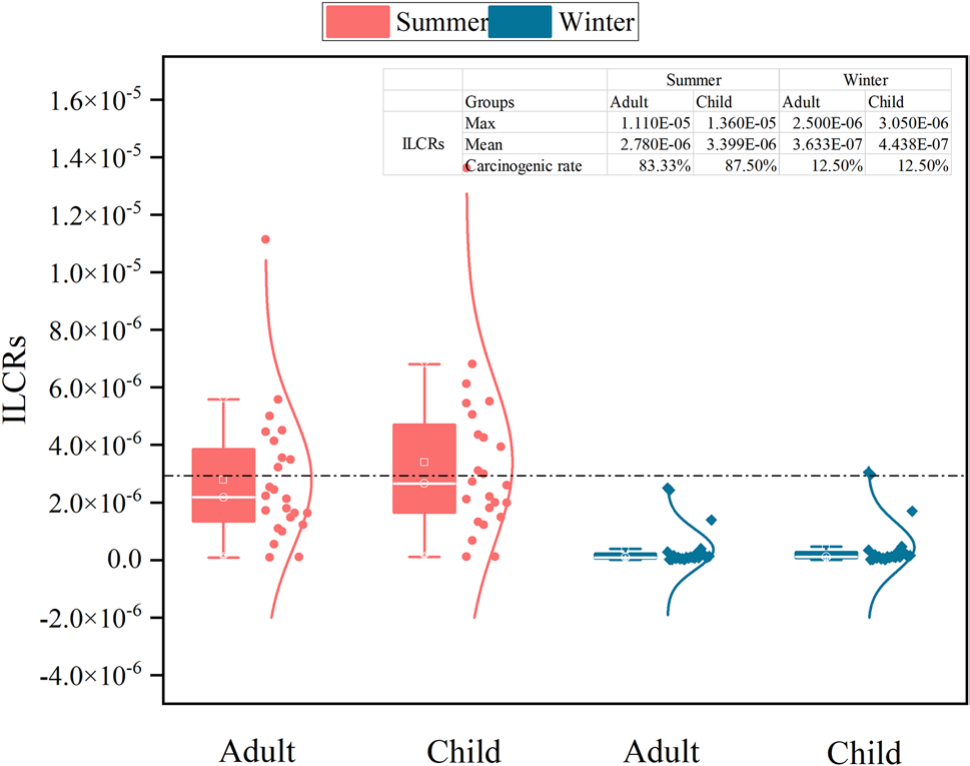
ILCRs value of PAHs in park soil.

As shown in Fig. 5, the mean values of ILCRs for adults and children regarding PAHs in the summer soil were 2.783 × 10^–6^ and 3.400 × 10^–6^, respectively. ILCRs in adult, 83.3% were greater than 10^–6^, and in children, 87.5% were greater than 10^–6^, reaching a low risk level. Among them, the maximum ILCRs in adults and children were greater than 10^–5^ at sampling point 3 of the Botanical Garden. The mean values of ILCRs for adults and children regarding PAHs in the winter soil were 3.632 × 10^–7^ and 4.439 × 10^–7^, respectively, which were lower than the minimum ILCRs. Only 12.5% of adult and child ILCR values greater than 10^–6^ reached a low risk level, and the sampling points were all in Toutunhe Park. In addition, we can observe that the carcinogenic risk of soil in the park is much higher in children than in adults. This is because children’s organs, nerves and immune systems may be more sensitive to pollutants, as children are developing; at the same time, children move easily on the ground and have greater access to contaminated soil. The carcinogenic risk of the summer soil to the human body was much higher than that of winter soil. This is inconsistent with the results of Fangfang et al.^18^. The reason may be that Urumqi is affected by the new coronavirus (COVID-19), and the amount of artificially produced PAHs decreased. The ILCRs in adults and children in both summer and winter are as follows: ingestion > dermal > inhalation. Compared with other exposure routes, the lifetime carcinogenic risk of inhalation is nearly negligible. This is because the lifetime carcinogenic risk caused by children's frequent hand-to-mouth contact while playing in the park is much higher than that of inhalation. The results show that the ILCRs of the PAH exposure pathway of soil samples from Urumqi parks in winter were all lower than the minimum acceptable risk level. The lifetime carcinogenic risk of adults and children is negligible, but high ILCRs in summer require particular attention.

The NIPI characterizes the impact or effect of soil pollutants. NIPI can be divided into five levels: NIPI is less than or equal to 0.7, where soil pollution is at a safe level; NIPI is equal to 1.0, where soil pollution is at the warning line; NIPI between 1.0 and 2.0, where soil pollution is at a weak level; NIPI between 2.0 and 3.0, where soil pollution is at a moderate level; and NIPI greater than 3.0, where soil pollution is severe^37^.

**Figure 6:**
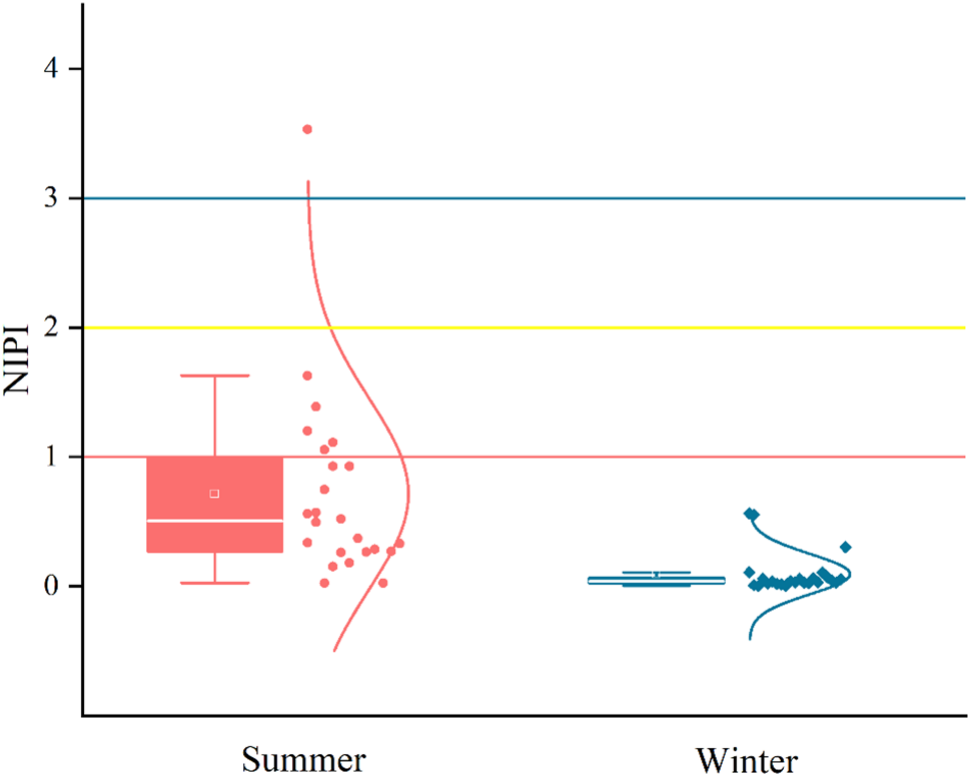
NIPI value of PAHs in park soil.

As shown in Fig. 6, in summer, the NIPI range of park soil in Urumqi is 0.025–3.532, with an average value of 0.715. The NIPI value of 75% of the sampling points was less than 1.0, and the ecological risk of the soil was safe. The NIPI value of 20.8% of the sampling points is between 1.0 and 2.0, mainly located in South Park, Zhiwu Park, Shihua Park and Toutunhe Park. The soils of these parks have more PAH accumulation factors than the soils of other parks. At the same time, the maximum value of NIPI is 3.532, which is a severe pollution level. This may be related to the pile of garbage in the park. The NIPI range of soil in Urumqi city parks in winter was 0.004–0.564, with an average value of 0.095. The NIPI value of all sampling points was less than 0.7, indicating that the ecological risk of soil in all parks in winter is at a safe level. The ecological risk of park soil in Urumqi in winter is much lower than that in summer.

In general, the ecological risks of soil in Urumqi parks can be ignored. Among them, South Park, Zhiwu Park, Shihua Park and Toutunhe Park should adopt a variety of control measures to reduce the ecological risk of soil PAHs through strict environmental management.

## Conclusion

In eight typical parks in Urumqi, the average concentration of PAHs in soil in the summer was 3.304 mg/kg. The average concentration of PAHs in soil in the winter was 0.644 mg/kg. The single PAHs in some soil sampling points in summer were higher than the national "Construction Land Standard" (GB36600-2018) soil pollution risk screening value. In summer, 91.6% of the soil samples were moderately or severely polluted, while 20.8% of the soil samples in winter were moderately or severely polluted. The average value of PAHs in soil in the park area in summer was 5–6 times that in winter. PAHs are mainly composed of high-ring PAHs from traffic sources, but the proportion of low-ring PAHs 2–3 increases significantly in winter. Compared with other regions, pollution is more serious, especially in the summer.

In summer, the plants with high PAHs content in leaves are short herbs, while in winter, they are tall arbors. The proportion of seven carcinogenic PAHs was higher in the roots than in the leaves. PAHs in 70% of plant leaf samples were higher in winter than in summer. PAHs are mainly composed of five rings in summer, from traffic sources, and three rings in winter, from the combustion of coal biomass. Compared with plants of other areas, pollution is lighter.

The TEQ values of soil PAHs in South Park, Zhiwu Park, Shihua Park and Toutunhe Park were higher. The summer soil of these four parks is at a low pollution level, compared with other parks. The mean ILCRs in adults and children in all parks reached a low-risk level in summer. The cancer risk of children in different seasons is much higher than that in adults. The sequence of ILCRs for different exposure routes is ingestion > dermal > inhalation, and the lifetime cancer risk of inhalation can be almost ignored.

## Materials and methods

### Overview of the study area and sample collection

**Figure 7:**
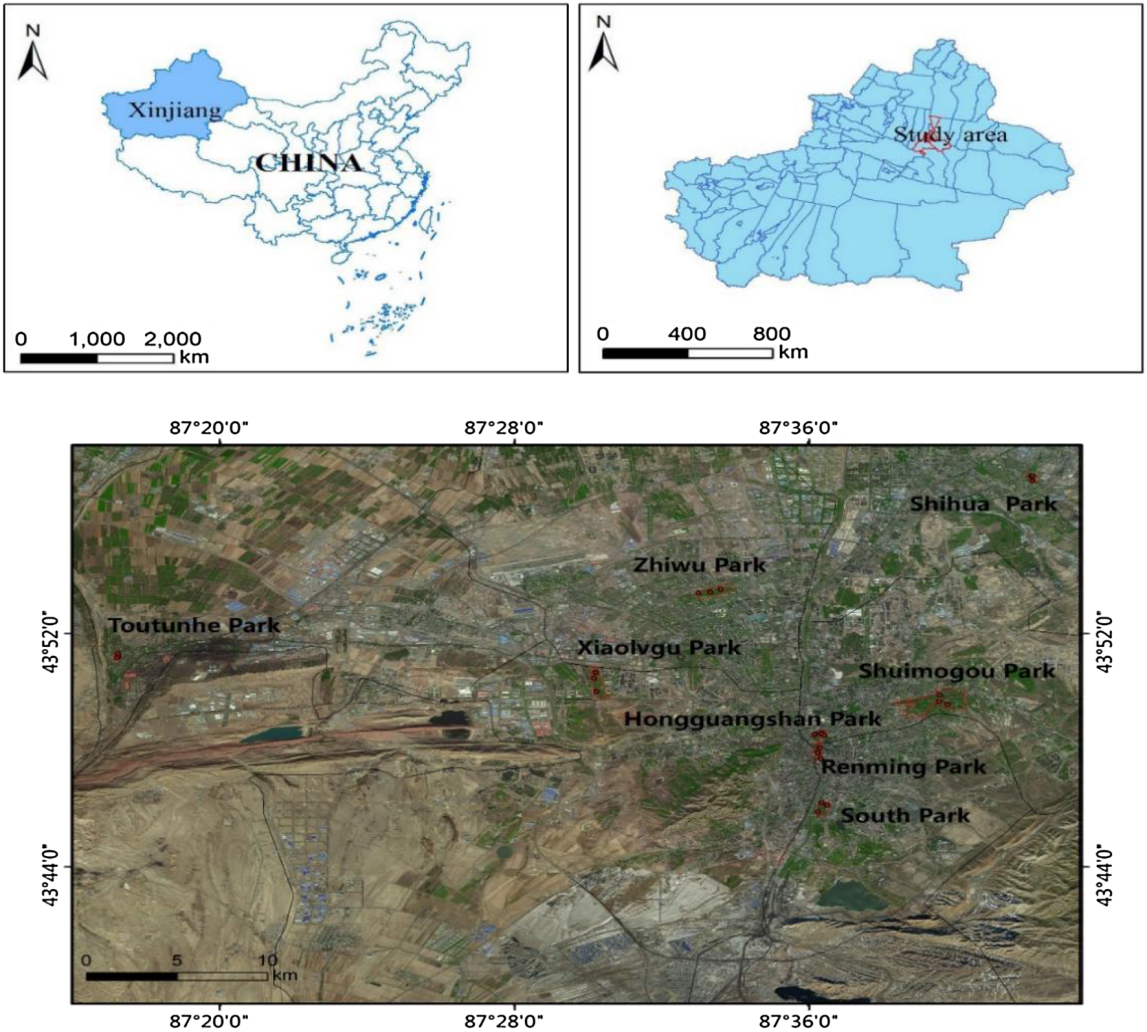
Map of sampling sites in parks in Urumqiy (The ArcGIS 10.8 was used to create the geographic map of the study area, URL: http://36.112.130.153:7777/DSSPlatform/index.html).

In this study, eight typical parks in Urumqi City were selected as the soil sampling sites. As shown in Fig. 7, these are: South Park, Shuimogou Park, Xiaolügu Park, Renming Park, Zhiwu Park, Shihua Park, Hongguangshan Park and Toutunhe Park. The sample collection periods were from November 20 to November 22, 2020 (winter), and from June 13 to June 14, 2021 (summer). According to the area of the park, nine sampling points were selected for each park, and every third soil samples were mixed into a mixed sample. The depth of sampling was 0–10 cm. In total, 48 surface soil samples were collected. Meanwhile, South Park was selected for the sampling of plants because it has a large number of plant species and higher passenger flow. Ten kinds of common plants in the park were selected, including five kinds of arbor plants, namely, longclaw willow, juniper, small leaf poplar, white elm and Platycladus orientalis, and five kinds of herbaceous plants, namely clover, silver edge grass, golden chrysanthemum, wild Artemisia and peony. Three plants with the same growth status were selected for each plant, and their leaves and roots were collected. The leaves and roots of each plant collected at different sampling points were mixed separately. There were 30 plant samples in total.

### Sample handling

We accurately weighed 15 g (accurate to 0.01 g) of soil samples and 1 g of plant samples after freeze-drying, grinding, and screening, put them into 250 ml and 50 ml centrifuge tubes, added 30 ml and 20 ml (v:v = 1/1) dichloromethane acetone solution, respectively, and allowed to stand for 2 h. Homogenates were extracted on the Beater (ULTRA-TURRAX) for 1 min, and then extracted in a water bath constant temperature (30 °C) oscillator (SHA-C) for 30 min. Subsequently, the mixture was centrifuged using a centrifuge (CT18RT) at a speed of 10,000 r/min for 5 min, and then 5 ml of upper organic phase clear liquid was taken. The mixture was concentrated at 40 °C with a parallel evaporator (BUCHI- Syncore), and the volume was combined with 2.5 ml of n-hexane, swirled for 15 s, and then passed through a 0.22 μm membrane injection bottle for testing.

### Test analysis

GC–MS analysis test conditions: gas chromatography conditions; injection temperature: 280 °C; no shunt; injection volume: 1.0 μL; column flow rate: 1.0 ml/min (constant flow rate); column box temperature: 60 °C. Mass spectrometry reference conditions electron bombardment source (EI); ion source temperature: 230 °C; ionization energy: 70 eV; interface temperature: 280 °C. Mass scanning range: 33–555 amu; solvent delay time: 4 min. Scanning mode: full scan mode (qualitative analysis) and selective ion SIM mode (quantitative analysis).

### Quality control

The quality of the sample was controlled by a blank test, recovery of the blank sample, and parallel sample. One blank sample was used in each batch of samples for the control. No soil or plants were added to the blank sample. Other reagents and treatment conditions were the same as those of normal samples, to ensure cleanliness and pollution-free instruments, reagents, and experimental containers. Each sample was analyzed using a double horizontal sample. The detection limits of 16 PAH monomers ranged from 1.3 × 10^–6^ to 8.5 × 10^–5^ mg/kg. Standards (200 μg/kg) of 16 PAHs at known concentrations were added to the uncontaminated matrix samples, and the actual concentrations were measured by the same pre-treatment and quantification method as the samples after 2 h of standing. The value of recovery rate was obtained from (Measured concentration of spiked matrix / spiked concentration)*100%. The experimental analysis shows that the recovery rate of the blank addition was in the range of 75.64–115.89%; there by meeting the requirements of Soil and sediment–Determination of polycyclic aromatic hydrocarbon by Gas chromatography–Mass Spectrometry Method (HJ 805–2016).

### Toxicity equivalent concentration method (TEQ)

The carcinogenicity and toxicity of benzo(a)pyrene (BaP) were the strongest among the seven carcinogenic PAHs. Usually, BaP is used to evaluate carcinogenic PAHs, and the toxic effect factor (TEF) is a parameter describing the carcinogenic ability of each monomeric PAH corresponding to BaP^38–40^. The formula for calculating the equivalent concentration of PAHs (TEQ) based on the toxic equivalent factor is as follows^40,41^:

1 $${\text{TEQ}} = \sum ({\text{PAH}}_{{\text{i}}} \times {\text{TEF}}_{{\text{i}}} )$$

where PAH_*i*_ is the content of PAH monomer *i*, TEF_*i*_ is the toxicity equivalent factor of PAH monomer *i*, TEQ is the toxicity equivalent of the equivalent concentration of the compound, and the TEF value of BaP is 1, which is the highest among all PAHs^42^. According to the toxicity equivalent of BaP, we used the toxicity equivalence factor (TEF) shown in Table 4 to convert the concentration of PAHs into the toxicity equivalent concentration of PAHs.

**Table 4:** Parameters used in lifetime carcinogenic risk assessment.

Exposure parameters	Unit	Children	Adult
Body weight (BW)	kg	15	60
Exposure frequency (EF)	d/year	250	250
Exposure duration (ED)	year	3	10
Air inhalation rate (IRair)	m^3^/d	5	15
Soil inhalation rate (IRsoil)	mg/d	400	200
Skin exposed area (SA)	cm^2^/d	1600	4350
Skin adhesion factor (AF)	mg/cm^2^	0.2	0.07
Skin adsorption parameter (ABS)	–	0.13	0.13
Average lifespan (AT)	day	70 × 365	70 × 365
Soil particle emission factor (PEF)	m^3^/kg	1.36 × 10^9^	1.36 × 10^9^

### Incremental Lifetime Carcinogenic Risks (ILCRs)

The incremental lifetime carcinogenic risk (ILCRs) of soil PAH exposure was evaluated according to USEPA standards^43^. Children and adults are exposed through three main exposure routes: ingestion, dermal and inhalation^44^. The ILCR equation for soil is as follows:

2 $${\text{ILCR}}_{{{\text{Ingestion}}}} = \frac{{{\text{CS}} \times \left( {{\text{CSF}}_{{{\text{Ingestion}}}} \times \sqrt[3]{{\frac{BW}{{70}}}}} \right) \times {\text{IR}}_{{{\text{soil}}}} \times {\text{EF}} \times {\text{ED}}}}{{{\text{BW}} \times {\text{AT}} \times 10^{6} }}$$

3 $${\text{ILCR}}_{{{\text{Dermal}}}} = \frac{{{\text{CS}} \times \left( {{\text{CSF}}_{{{\text{Dermal}}}} \times \sqrt[{3}]{{\frac{{{\text{BW}}}}{{{70}}}}}} \right) \times {\text{SA}} \times {\text{AF}} \times {\text{ABS}} \times {\text{EF}} \times {\text{ED}}}}{{{\text{BW}} \times {\text{AT}} \times {10}^{{6}} }}$$

4 $${\text{ILCR}}_{{{\text{Inhalation}}}} = \frac{{{\text{CS}} \times {\text{(CSF}}_{{{\text{Inhalation}}}} \times \sqrt[{3}]{{{\raise0.7ex\hbox{${{\text{BW}}}$} \!\mathord{\left/ {\vphantom {{{\text{BW}}} {{70}}}}\right.\kern-\nulldelimiterspace} \!\lower0.7ex\hbox{${{70}}$}}}}{)} \times {\text{IR}}_{{{\text{air}}}} \times {\text{EF}} \times {\text{ED}}}}{{{\text{BW}} \times {\text{AT}} \times {\text{PEF}}}}$$

5 $${\text{ILCR}} = {\text{ILCR}}_{{{\text{Ingestion}}}} + {\text{ILCR}}_{{{\text{Dermal}}}} + {\text{ILCR}}_{{{\text{Inhalation}}}}$$

where CS is the toxic equivalent concentration of monomeric PAHs in the sample (mg/kg), 10^6^ is the conversion coefficient of PAH concentration, and CSF is the carcinogenic slope factor (mg/(kg·d)), based on the carcinogenic ability of BaP. The CSF_Dermal_, CSF_Ingestion_, and CSF_Inhalation_ are 25, 7.3, and 3.85 (mg/(kg·d)), respectively^44^. Other parameter values were obtained from the Guidelines for Site Environmental Assessment (DB11/T 656-2009), as shown in Table 4.

### Nemerow multifactor pollution index (NIPI)

The Nemerow multifactor pollution index (NIPI) is one of the most widely used indicators to reflect the ecological risk of soil pollutant exposure^37^. It can characterize the role of soil pollutants in a seriously polluted environment. The calculation method for the NIPI is as follows:

6 $${\text{P}}_{{\text{i}}} = \frac{{{\text{C}}_{{\text{i}}} }}{{{\text{C}}_{{\text{s}}} }}$$

7 $${\text{NIPI}} = \sqrt {\frac{{{\text{P}}_{{{\text{iavg}}}}^{{2}} + {\text{P}}_{{{\text{imax}}}}^{{2}} }}{{2}}}$$

where C_*i*_ is the measured concentration of monomer *i*, C_*s*_ is the soil environmental quality standard value of monomer *i*, and Pi is the pollution index of PAH monomer *i* in the soil. P_iavg_ is the arithmetic mean of PAH pollution index of each monomer, P_imax_ is the maximum value of the PAH single factor pollution index at the *i*th sampling point, and NIPI is the multi-factor pollution index at the sampling point. This study used the class II standard value in the soil environmental quality standard (GB15618-2008).

The use of plants in the present study complies with international, national, and institutional guidelines, the collection of plant samples was done with the consent of local relevant departments and institutions.

## Data Availability

Data may be obtained from the authors upon request.
